# Mr. Hume, on the Pharmacopœia

**Published:** 1804-04-01

**Authors:** J. Hume

**Affiliations:** Long Acre


### *To the Editors of the Medical and Physical Journal.*

GENTLEMEN,

IT is impossible, I believe, for any candid observer to peruse our present metropolitan pharmacopœias, without deploring the great dissimilarity so conspicuous, not only in the nomenclature, the arrangement, the choice of *formulæ*,

*formulæ*, but often, which is the most material fault, in the proportion of an essential article in the same identical compound. Whether we compare the pages with each other in either volume, or contrast the dispensatories themselves, we cannot but lament the great disparity; the want of precision, uniformity, and simplicity; and that, in respect to nomenclature, there should be so much dissonance of opinion on what ought to be retained and what rejected.

On this subject, which may, perhaps, be properly termed *national* pharmacy, much remains to be done; reform, and very great reform indeed, seems still wanting to render a pharmacopœia more generally acceptable, free from errors, and divested of those numerous ambiguities which disgrace its pages; in short, a pharmacopœia truly deserving the epithets *Imperialis Britannica*. I fear, however, unless a deputation from each college be authorised to take upon them to execute this important work, we must in vain look for unanimity and for that degree of improvement which, from the late rapid advances in chemical knowledge, we have a right to expect; and which, on account of the present happy union of these realms, seems now loudly demanded.

My motives for addressing to you the following observations, arise more from a wish to prompt some of your numerous correspondents, whose time and abilities render them more capable to take up this subject, and point out what ought to be done, and how best effected, than from conviction of my own competency. It may, however, be expected that I should accompany this general disapprobation with some reasons or proofs on which it is founded. I shall, therefore, for the present, content myself with offering a few examples, and in a very unmethodised and summary way, point out what I conceive to be culpable, leaving the subject still open to any future or more extended discussion. As the greater part of the objections are to be deduced from the *Pharmacop. Edinb.* it may be proper, once for all, to declare, that I have selected this work in consequence of its having been published at the latest period, and, therefore, more to be depended upon for perfection: I am also induced to make this choice from a firm belief that the College of Edinburgh stands second to no other in reputation, more especially for every branch of medical science.

In the Ph. Edinb. we find the same name sometimes put adjectively, at others as a substantive, and without any apparent apparent intention; on the contrary, for obvious reasons, rather liable to lead into error. We observe *opium, opiatum*—*scilla, scillitica*—*camphora, camphoratum*—*ammonia, ammoniatum*. The adjective *compositum* is applied at random in all its cases. Why add it to *tinct. cassiæ sennæ* and not to *tinct. mimosæ catechu*? Why tack it to *pilulæ rhei*? There is no other *formulæ* with which these may be confounded. *Compositum* is, nevertheless, often omitted where it ought to have been added. It seems a general resolution to apply the Linnæan names, together with all their systematic, generic, and specific expletives and appendages. This, I am fully persuaded, was never intended by that celebrated author; any farther than in systems of botany, natural history, and in a few other similar productions, these names seem inadmissible. In every *materia medica*, they ought to stand first among the *synonima*; and in determining the proper title for each *formula*, that name should be chosen which is short, free from ambiguity, and that has been, and is likely to remain, most universally intelligible. There is certainly as much impropriety in discarding *alumen, olibanum, minium, lithargyrus, cerussa, nitrum, galbanum, cicuta, cinnabaris, calamine*, and an infinite number of simple names, with which we are well acquainted, as it would be were the geographer to expel the word *ocean* or *sea* out of his vocabulary, and substitute *solution of muriate of soda, magnesia*, &c. or the physician who would expunge the word *water*, and order his patient to be immersed in some warm *oxyde of hydrogen*. The new chemical nomenclature is certainly inadmissible, in its full latitude, into pharmacy; we may, no doubt, take great advantages without rendering the titles of the necessary compositions so excessively verbose, so periphrastic, and full of circumlocution.

*Gummi Arabicum* is now changed for *gummi mimosæ niloticæ*, as if any danger could arise from retaining the former. *Gummi* is retained in the *emulsio gummi mimosæ niloticæ*, and omitted in the *mucilago*. Another formula, having the same ingredient for its basis, is named trochisci *gummosi*; and we have also, strange to tell, *emplastrum gummosum*: this, however, contains no *gum. mimos. nilot.* but is composed of empl. litharg. *gummi resina ammoniaci*, and *gummi resina bubonis galbani*.

The pilulæ *opii* of the Ph. Lond. may be said to be the proper parallel to pilulæ *opiatæ* of the Ph. Edin. but the former contains one-*fifth* of its weight of opium, and the latter only one tenth! It might appear invidious were

 I to

I to say which College is to blame for not correcting this dangerous inequality; there should be no disparity in a composition of such moment, and of such consequence to the lives of the inhabitants in all the sister kingdoms, from all parts of which prescriptions are reciprocally and constantly sent. *Pilulæ hydrargyri* of both the colleges are in the same predicament, in respect to the proportion of the mercury; that of the London Disp. contains one-*third* of its weight; that of the Edinb. Coll. one-*fourth*; and the Dublin Coll. perhaps about a *fifth*; for this is by no means an accurate prescription. As to the pills being divided, it is absurd and quite unnecessary; the formula adopted by the two former colleges, provided the conserve be made with proper sugar, will keep very long without changing and turning hard, as has been alleged.

The conjunction *et* and preposition *cum* are put in several instances, very ambiguously. In some formulæ they mean a chemical combination; in others a mere mixture, as if the ingredients had been mixed together in a mortar or with a spatula. Thus *pulvis ipecac. et opii, pilulæ aloës cum colocynthide:* these are mixtures in which no chemical change has taken place. But we have *sulphas potassæ cum sulphure,* where a perfect *chemical* combination has taken place, and will prove to be completely distinct from a mixture of sulphur and sulphate of pot-ash made by stirring the two ingredients together. By the way it is proper to add, that this title is not well adapted, for it will be found to be a *sulphite,* not sulphate of potash; its solubility, the form of crystals, and other distinctive marks, will demonstrate that the sulphureous acid is chiefly combined with the pot-ash. This process is very ancient, though rather clumsy, if a perfect *sulphite* be required; nevertheless, if the medicine be useful, as I have reason to believe it is, it ought not to be discarded.

*Oxidum antimonii cum phosphate calcis.* Are we well assured that a mixture of these two substances form exactly what we expect, and is there no better or more explicit title for this powder? Would the *pulvis algarothi,* which I apprehend to be the most uniform oxyde of this metal, joined to phosphate of lime, produce the same medicament as the *pulv. antimonialis* Ph. Lond.? Till these questions be answered we had better remain contented with the *name* as well as the mode of preparing the *pulv. antimonialis.*

Why is *flavus* tacked to *sub-sulphas hydrargyri*? There is no other prescribed. Perhaps *hydrarg. flavus* would have been unobjectionable. Indeed the *sub* and *super* are very very objectionable expletives, and, I humbly conceive, quite unnecessary in pharmaceutic names. We had better recur to old arbitrary names, some of which we still retain, such as *alcohol*, &c. &c. &c.

In a former paragraph I ought to have added, that as the Edinburgh College employ *opium* only in its *crude* state; and the London and Dublin Colleges have uniformly ordered opium *purified*, it will in all cases be requisite, in making comparative estimates of the relative strength of any formula, to recollect that the proportion in power is as 9 : 12.

*Emplast. simplex* contains one-fourth of its weight of *resin*, and the *emplast. resinosum* only one-sixth; it would have been more consonant to have named the first empl. *ceræ*, as in the Ph. Lond. and the latter empl. *adhæsivum*; for we may find many instances where the names are chosen more from the *quality* than the component ingredients of the mixture, such are *elect. aromat. pulv. aromat. trochis. gummosi*, &c. in Ph. Edinb.

*Emplastrum oxidi plumbi semivitrei*, when contrasted with *emplastrum lithargyri*, seems frightfully verbose. Surely the latter cannot be objectionable nor cause even a shadow of doubt. There is certainly too much play upon the word *oxidum* through the whole of the *Ph. Edinb. Cerussa* is distinguished as *oxidum plumbi albi*, whereas it chiefly consists of *carbonas* plumbi.

The word *impurum* stands frequently with an ill appearance, and very ungracefully. Why admit any thing *impure* when it is in our power to have it perfect. *Oxidum zinci impurum, carbonas zinci impurus*, and there might have been added some other *impura*, for, in the chemical department, they now and then occur. It has been lately shewn by an analysis, published I believe in the *Philos. Trans.* that there are many varieties of *calamine*; if so, why admit *carbonas zinci impurus*?

*Sulphas zinci*, as it now stands, must contain excess of acid enough to keep also iron in the solution, with which the zinc is constantly found united; and, as if the impurity should not be lost, there is an addition of acid in the subsequent formula, *solutio sulph. zinci, solutio acetitis zinci.* However carefully and accurately this may be prepared, an addition of acetite of barytes will demonstrate the presence of sulphuric acid. Sulphate of lead has been deemed soluble in particular circumstances, so it is mentioned in the *Philosoph. Journal.* In the above solution I suspect the sulphuric acid remains in the solution in composition position with some of the zinc, so that this solution is *acetite* of *zinc*, some *sulphate* of *zinc*, and dilute acetous acid. As *acetite* of *zinc* is certainly an excellent topical remedy, the above formula should be corrected. Why not, at once, dissolve pure oxide of zinc in acetous acid, and either evaporate or add distilled water, according to the desire of the prescriber. In all our dispensatories, very little attention seems to have been bestowed on the *materia chirurgica*.

What is called *oxidum arsenici* in the list of *materia medica*, is certainly arsenious *acid*, as a solution of it in distilled water tinges litmus paper red.

*Tinctura ferri muriati* should never be made with *green* muriate, which must be the case if oxidum ferri *nigri* be employed.

Is there any sufficient reason for retaining both carbonas ferri and carbonas ferri *præcipitatum?*

*Tartris antimonii* is not, as the title imports, a combination of the metal with the *acid* of tartar; for Bergman has shewn that there must be some potash to form the *emetic tartar*, as it has been very properly termed by our predecessors.

*Ammoniaretum cupri* may, as a medicine, deserve its place in a Pharmacopœia, but not the appellation here bestowed upon it. Besides the *sulphate* of *ammonia*, which must remain in the mixture, there is also carbonic acid, either attached to the copper or ammonia, or, probably, to both.

If *sub* be properly added to *acetis cupri*, why is it not to *acetis plumbi?*

In giving names to the *formulæ*, I am inclined to suppose that the colleges either had in view, or might have availed themselves of, some uniform adherence to general principles in forming a Nomenclature, and, by using some words adjectively or substantively, thereby more explicitly denote what is meant. In general, the classes of *tincturæ, aquæ, syrupi, pilulæ, &c.* when the second substantive is in the *genitive case*, for the most part we may conclude that a *part* only of such article is in solution, though that is at the same time supposed to be the *virtue* or *essential* part. Thus, *tinct. cinchonæ, rhei, &c. infus. sennæ, gentianæ, &c.* only a *part* of the several ingredients is taken up by the menstruum; and this rule might be rendered general. On the contrary, it may be seen that this mode of particularising has not obtained even in such instances where it was very appropriate. We have in the same volume, *alcohol ammoniatum*,

*ammoniatum*, and *aqua ammoniæ, aqua potassæ, aqua calcis;* in all of these the whole of the article is in solution. Again, there is an *oleum ammoniatum*; now if we make it, as its title indicates, a parallel to *alcohol ammoniatum*, we shall discover a material difference; in the *alcohol* the whole of the ammonia is combined, but in the *oleum* there is superadded a quantity of water. Perhaps *potassa aquosa* would be more suitable, and calx aquosa, &c.; but these suggestions are not within my present aim.

I am, &c.

